# Possible relation between maternal consumption of added sugar and sugar-sweetened beverages and birth weight – time trends in a population

**DOI:** 10.1186/1471-2458-12-901

**Published:** 2012-10-24

**Authors:** Jacob Holter Grundt, Jakob Nakling, Geir Egil Eide, Trond Markestad

**Affiliations:** 1 Department of Pediatrics, Innlandet Hospital Trust, Lillehammer, Norway; 2 Department of Obstetrics and Gynecology, Innlandet Hospital Trust, Lillehammer, Norway; 3 Centre for Clinical Research, Haukeland University Hospital, Bergen, Norway; 4 Department of Public Health and Primary Health Care, University of Bergen, Bergen, Norway; 5 Department of Research, Innlandet Hospital Trust, Brumunddal, Norway; 6 Department of Clinical Medicine, University of Bergen, Bergen, Norway

**Keywords:** Pregnancy, Body mass index, Weight gain, Birth weight, Macrosomia, Large for gestational age, Glucose, Sugar, Sugar-sweetened beverages, Norway

## Abstract

**Background:**

High birth weight (BW) is a risk factor for later obesity. In Norway, mean BW and proportion of large newborns increased from 1989 to 2000 and subsequently decreased to the 1989 level by 2010. The purpose of the study was to explore causes of this temporary increase.

**Methods:**

From a regional prospective database pregnancy and newborn data were extracted for all 33088 singleton pregnancies resulting in live infants born at term without malformations during 1989–2010. Trends in BW, ponderal index and proportion of large newborns were related to individual prenatal exposures, including pre-pregnancy body mass index (PP-BMI) and gestational weight gain (GWG) for the years 2001–2010, and thereafter related ecologically to national population data on consumption of nutrients and physical activity.

**Results:**

For the regional cohort mean (standard deviation) BW increased from 3580 (453) grams in 1989/90 to 3633 (493) grams in 2001/02 (p<0.001), and decreased to 3583 (481) grams in 2009/10 (p<0.001). The proportion with BW>4500 grams increased from 2.6% to 4.8% (p<0.001) and subsequently decreased to 3.3% (p=0.002). The trends remained after adjustment for relevant exposures. For the years 2001/02 to 2009/10 (n= 15240) mean (SD) PP-BMI increased from 24.36 (4.44) to 24.85 (5.02) kg/m^2^ (p<0.001) while GWG decreased from 14.79 (5.85) to 13.86 (5.79) kg (p<0.001). The estimated net effect of changes in PP-BMI, GWG and other known exposures was a 6 grams reduction in BW from 2001/02 to 2009/10, leaving 44 grams reduction unexplained. National consumption of major nutrients did not change, but consumption of sucrose, in large part as sugar-sweetened beverages (SSB) changed in parallel to the BW trends.

**Conclusion:**

The temporary increase in BW and large babies in the regional cohort was identical to that reported for Norway. Individual level data on known pregnancy related predictors for BW could not explain these changes, but the parallel time trend in national consumption of sucrose, in particular as SSB, may lend support to a hypothesis that intake of sugar may have a direct effect on BW and infant body proportions independent of effects through PP-BMI and GWG.

## Background

The rapidly increasing prevalence of overweight and obesity (OWOB) is of major health concern, and all relevant risk factors need to be addressed in order to curtail this epidemic. High birth weight (BW) is associated with increased risk of later OWOB [1], and a suggested mechanism is fetal programming following intrauterine overnutrition [2,3].

Data from the Medical Birth Registry of Norway (MBRN) revealed a temporary increase in mean BW (50 grams) and proportion of newborns with BW above 4500 grams (50 % increase, from 3.2 to 4.9 %) from 1990 to 2000, followed by a corresponding decrease during the years 2000–2010 [4,5]. The proportion of newborns with BW under 2500 grams was unchanged. The same development in BW was also observed in our regional pregnancy and birth registry.

The aim of the present study was to assess possible causes of the temporary increase in BW and proportion of large babies. The effects of potential predictors on BW were assessed in a representative regional cohort of pregnant women in Norway, using multiple regression analyses. In addition, the development in BW was related ecologically to population level national data on nutrient consumption and physical activity. The hypothesis was that the temporary increase in BW could not fully be explained by changes in obstetric practices or maternal behavior known to influence BW, such as smoking and maternal weight.

## Methods

### Regional cohort

Since 1989 information on all pregnant women who gave birth at the two only obstetric departments in Oppland County, Norway, and their newborns were prospectively and routinely registered in a local countywide database, which thus covers nearly all births in the county. Expected date of delivery (EDD) was assessed by routine ultrasound at 17–18 weeks’ gestation. Demographic, behavioral and medical information were systematically collected at this examination and at birth. At birth, data registered in the standard pregnancy health record, which is updated for every woman on each prenatal visit in primary and specialty pregnancy care, were added to the register. Registration of maternal height, pre-pregnancy weight (PPW) and gestational weight gain (GWG) started from 2001.

**Figure 1:**
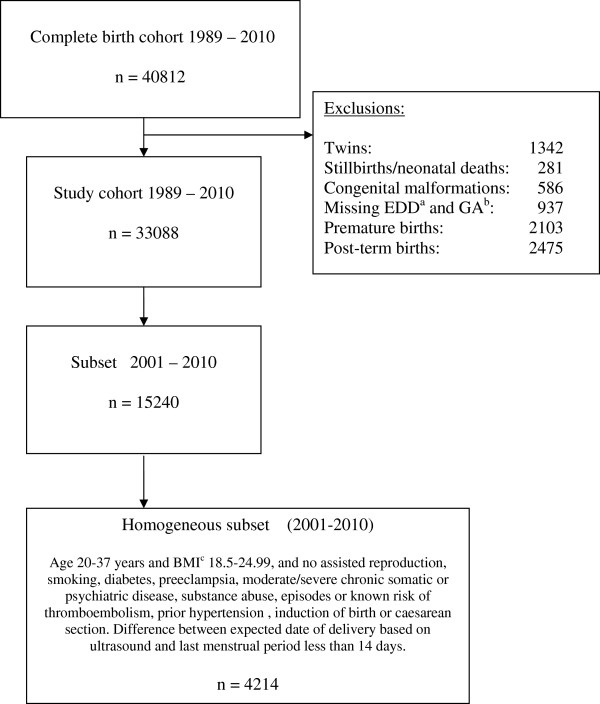
**Characteristics of the regional cohort.**^a^ Estimated date of delivery; ^b^ Gestational age; ^c^ Body mass index.

For the present analyses, mother-child dyads of singleton live born infants without malformations born at term (37–42 completed weeks) were extracted from the database (Figure 1). Analyses were also performed on a selected group of normal pregnancies and deliveries (*homogeneous subset*) for the years 2001–2010, i.e. age 21–37 years, pre-pregnancy body mass index (PP-BMI) 18.5-24.99, no smoking or substance abuse, no assisted fertilization, no diseases or pregnancy complications, spontaneous onset of labor and vaginal delivery (Figure 1). The purpose of also performing analyses in the subset was to reduce possible confounding, for example by time trends in obstetric practices.

Variables assumed to be of potential importance for BW and crown-heel length were extracted from the database. Gestational age at birth was assessed on basis of the ultrasound examination at 17–18 weeks' gestation if performed, otherwise on last menstrual period. The same methods were adhered to for the whole 22 year period [6]. PPW and maternal height were registered if obtained within the first 8 weeks of pregnancy. GWG was the difference between the weight obtained on admission for delivery and the PPW. Preeclampsia was defined as diastolic blood pressure > 90 mmHg and proteinuria, and gestational diabetes as fasting blood glucose > 6.1 mmol/l or 2-hour-glucose-level > 7.8 mmol/l on an oral glucose tolerance test. BW and crown-heel length were recorded immediately after birth. Macrosomia was defined as BW above 4500 grams. Ponderal index (PI) of the baby was calculated as BW (kg) divided by the third power of crown to heel length (m^3^) [7].

### National data on nutrition and physical activity

Estimates of nutritional intakes through the study period were based on reports from the Norwegian Directorate of Health [8] describing trends in consumption of energy and nutrient groups in the population. These reports are based on yearly consumer registrations (all food and drinks purchased by a nationally representative selection of households) conducted by Statistics Norway, on results from other specified population surveys, and on food industry sales data. All sugars are imported, and estimates of annual per capita gross consumption of added sugars in the data from the Norwegian Health Directorate, included that of SSB, were based on reports of sugar imports.

The Statistics Norway registrations do not include out-of-home consumption, and soft drinks are underreported [9], [10]. Consumption of sugar-sweetened beverages (SSB) was therefore based on national sales figures reported by the organization of industrial beverage producers in Norway [10], [11]. The assumption that pregnant women consumed SSB similar to the national average is supported by studies on Norwegian pregnant women during the studied period [12,13].

Norway has small regional differences in socioeconomic characteristics, and the regional cohort (Oppland county) scores close to the Norwegian average on public health statistics. The appropriateness of estimating overall nutrition on national data are further supported by a comprehensive national nutritional survey [14] which showed that intakes based on detailed registrations by a representative proportion of the population was similar to that reported from the Statistics Norway surveys. Furthermore, the Norkost study [14] showed small differences in intake of most main food and nutrient groups between regions and socioeconomic groups, and the nutrient intakes of 20–40 year old women were similar to that of pregnant women in a nationwide study [13] except for a somewhat higher intake of energy, and carbohydrates in terms of cereals, fruits and fruit juice among the pregnant women.

Estimates of trends in physical activity were based on a biannual nationally representative surveys of self-reported activity for 20–40 year old women for the years 1989–2009 ([Norwegian Monitor 1989–2009, Synovate], Gunnar Breivik, personal communication, 2011), and trend estimates for work related physical activity were based on a study combining fifty-two Norwegian population studies [15].

### Data analyses

Since BW parameters increased until 2000/01 and subsequently decreased, and since registration of maternal PP-BMI and GWG started from 2001, the two time periods 1989–2000 and 2001–2010 were first compared for potential explanatory and outcome variables using t-tests and chi-square tests as appropriate. Each period was then assessed separately for time trends using simple regression analyses; linear regression analysis for continuous and logistic regression analysis for dichotomous variables. For continuous response variables the estimated effect sizes are presented as regression coefficients (B-values) with 95% confidence intervals (CIs), e.g. the change in BW per one unit change in predictor, and the explained variance for a model (adjusted R^2^) is given. For dichotomous response variables the estimated effect sizes are presented as odds ratios (ORs) with 95% CIs.

To assess possible non-linear trends spanning the 1989–2010 period, and comprehensively adjust for effects of individual variables on BW and PI, we used multiple fractional polynomial model regression analysis (MFPR) [16]. Variables with a plausible explanatory potential were first assessed for correlation with BW. Those with a correlation coefficient higher than 0.025 (Spearman rho) and a significance probability (p-value) less than 0.1 were initially included. “Year of birth” (YOB) values were transformed to a range from 0 to 21. Thereafter the MFPR models were developed with variables being selected and kept in the model using a stepwise process. P-values ≤ 0.05 were considered significant. Missing data were excluded listwise in the regression analyses. MFPR was also performed separately for the period 2001–2010 to evaluate effects of including PP-BMI and GWG as explanatory variables. Binary logistic regression was performed to assess the adjusted effect of YOB on macrosomia rate for 1989–2000 and 2001–2010 separately.

Adjusted BW estimates isolating the effects of YOB on BW and PI trends were calculated using MFPR models. This procedure isolates the effect of an individual predictor, ”partial prediction”, by setting the values for all other predictors to the lowest alternative for dichotomous variables, and to the population mean for continuous variables. In MFPR models adjusted effect estimates for a continuous predictor can be modeled in terms of fractional polynomials, and may therefore vary non-linearly. If adequate, multiple linear regression analyses were performed to simplify presentation of effect estimates for continuous predictors.

Net effects of significant exposure variables on change in BW from 2001/02 to 2009/10 were estimated. For continuous variables effect sizes from multiple regression analyses were multiplied by actual changes in mean values. For dichotomous variables the change in number of exposed subjects (calculated as the mean number per year for the years 2009/2010, i.e. 1650, multiplied by the percent change in number exposed) was divided by the mean number of births in 2009/10 (n = 1650) and multiplied by the effect size of the predictor.

Statistical analyses were performed using SPSS version 15.0.1 (SPSS Inc; 2006), except for the MFPR analyses which were performed using Stata version 12 (Stata Corp LP; 2011).

**Figure 2:**
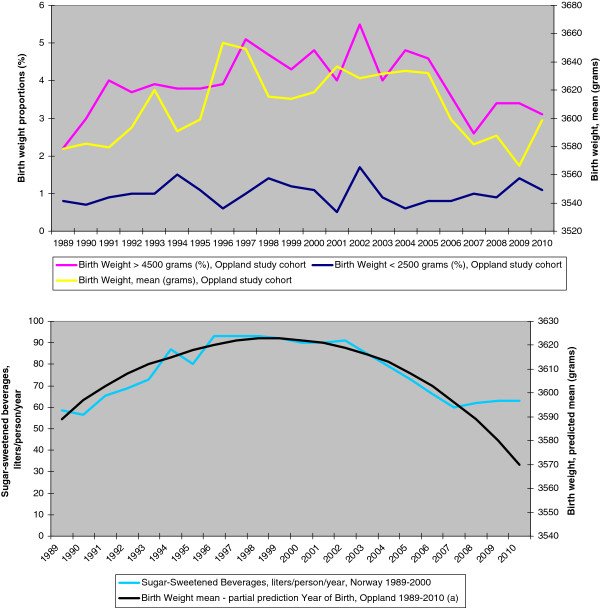
**Time trends in birth characteristics and consumption of sugar-sweetened beverages.** Changes in mean birth weight, proportions of large and small newborns in the regional cohort (upper panel), and adjusted birth weight estimate (partial prediction “Year of birth”) in the regional cohort shown together with national consumption of sugar-sweetened beverages (lower panel). ^a^ "Partial prediction Year of birth, Oppland 89-10" was obtained from the 1989-2010 Multiple Fractional Polynomial Regression (MFPR) model for BW (see method section).

Statistical analyses were only performed on data from the regional cohort. Estimates of nutritional intakes and physical activity were based on the national databases and described in terms of time trends and visualized graphically and in tables (e.g. Figure 2, lower panel), thus representing an ecological approach.

The regional database was established as a health care quality control register supplementing ordinary patient records. Anonymous data was extracted for analyses. Research on anonymous registry data is routinely exempt from ethical review and informed consent requirements by the ethics research committees in Norway.

## Results

### Regional cohort characteristics and trends

For the years 1989–2010, 40812 mother-infant dyads were registered in the database accounting for 99.9 % of those registered for the county in the MBRN. Of these, 97% were Caucasians and 33088 (81%) were eligible for this study (Figure 1). Of the 15240 dyads included during 2001–2010, 4214 (27.7%) constituted the homogeneous subset. EDD was based on ultrasound assessment for 98.8%.

Proportions of pregnancies with diabetes, preeclampsia, induction of labor and caesarean sections increased while the proportion of smokers decreased during the study period (Table 1). Other trends with possible significance for BW showed partly opposite directions (Table 1). From 1989/90 to 2001/02 the mean (standard deviation (SD)) BW increased from 3580 (453) grams to 3633 (493) grams (p<0.001), and subsequently decreased to 3583 (481) grams in 2009/10 (p<0.001, Figure 2). The macrosomia rate increased from 2.6% to 4.8 % (p<0.001) and subsequently decreased to 3.3 % (p=0.002, Figure 2). Mean PI showed the same trend. Trends in BW, PI and macrosomia did not change after adjusting for exposure variables known for the whole period using MFPR and logistic regression. The proportion of newborns with BW < 2500 grams did not change during the period. The adjusted effect of YOB on BW remained curvilinear (Figure 2), modeled as the following partial predictor

(1) $$\begin{array}{l} 3622.199+61.91863\left(\sqrt{\frac{YOB+1}{10}}-1.101\right)\\ \;-8.524001\left({\left(\frac{YOB+1}{10}\right)}^{3}-1.783\right)\text{.} \end{array}$$

**Table 1:** Pregnancy and newborn characteristics of the regional cohort (Oppland county) born at term from 1989 to 2010

**Response variable**	**Births 1989–2000(n = 17848)**^**a**^		**Births 2001–2010(n = 15240)**^**b**^		**Comparing**^**j**^**89-00**^**a**^**vs 01-10**^**b**^	**Homogeneous subset**^**d**^**2001–2010(n = 4214)**^**c**^	
*Continuous variables*	**Mean (SD)**	**B (per year)**^e^**(95 % CI)**	**Mean (SD)**	**B (per year)**^e^**(95 % CI)**	**p-value**	**Mean (SD)**	**B (per year)**^e^**(95 % CI)**
Maternal age (years)	28.1 (4.9)	0.16 (0.13, 0.18) **	29.4 (5.1)	0.03 (0.00, 0.06) *	0.001	29.1 (4.2)	−0.03 (−0.07, 0.02)
Maternal height (cm)	na^f^	-	167.5 (6.1)	−0.02 (−0.06, 0.01)	na^f^	167.8 (6.1)	−0.06 (−0.00, -0.13) *
Pre-pregnant BMI^g^ (kg/m^2^)	na^f^	-	24.7 (4.8)	0.07 (0.04, 0.10) **	na^f^	22.0 (1.7)	−0.01 (−0.03, 0.01)
Gestational weight gain (kg)	na^f^	-	14.1 (5.7)	−0.12 (−0.08, -0.16) **	na^f^	14.7 (4.7)	−0.11 (−0.06, -0.16) **
Gestational age (days)	279.6 (7.8)	0.10 (0.06, 0.13) **	279.5 (7.9)	−0.12 (−0.08, -0.16) **	0.522	280.2 (7.3)	−0.15 (−0.08, -0.22) **
Birth weight (gram)	3611 (487)	4 (2, 7) **	3609 (485)	−7 (−4, -10) **	0.687	3607 (435)	−12 (−7, -16) **
Birth length (cm)	50.8 (1.9)	−0.01 (−0.01, -0.02) **	50.6 (1.9)	−0.01 (−0.00, -0.02) *	< 0.001	50.6 (1.8)	−0.02 (−0.00, -0.04) *
Ponderal index (kg/m^3^)	27.6 (2.3)	0.06 (0.05, 0.07) **	27.8 (2.4)	−0,04 (−0.02, -0.05) **	< 0.001	27.7 (2.3)	−0.05 (−0.03, -0.08) **
Head circumference (cm)	35.3 (1.4)	−0.01 (0.00, -0.01) *	35.5 (1.4)	0.04 (0.04, 0.05) **	< 0.001	35.4 (1.3)	0.03 (0.02, 0.04) **
*Binary variables*	**Prevalence (%)**	**OR (per year)**^h^**95 % CI**	**Prevalence (%)**	**OR (per year)**^h^**95 % CI**	**p-value**	**Prevalence (%)**	**OR (per year)**^h^**95 % CI**
Multiparity	60.2	1.01 (1.00, 1.02) **	59.8	0.98 (0.97, 0.99) **	0.486	59.0	0.96 (0.94, 0.98) **
Teenage mother	2.7	0.94 (0.91, 0.96) **	2.3	0.98 (0.94, 1.01)	0.014	na^f^	-
Smoker	25.1	0.98 (0.97, 0.99) **	13.8	0.91 (0.89, 0.92) **	< 0.001	na^f^	-
Diabetes	0.6	1.00 (0.95, 1.07)	1.7	1.13 (1.08, 1.18) **	< 0.001	na^f^	-
Preeclampsia	2.9	1.14 (1.10, 1.17) **	4.7	1.00 (0.97, 1.03)	< 0.001	na^f^	-
Birth after 40 weeks GA^i^	53.3	1.03 (1.01, 1.04) **	53.2	0.97 (0.96, 0.98) **	0.821	58.1	0.95 (0.93, 0.97) **
Induction of labour	4.6	1.16 (1.13, 1.19) **	13.7	1.09 (1.07, 1.11) **	< 0.001	na^f^	-
Caesarean section	10.0	1.03 (1.02, 1.05) **	13.9	1.02 (1.00, 1.03) *	< 0.001	na^f^	-
Maternal BMI < 18.5 kg/m^2^	na^f^	-	3.4	0.99 (0.96, 1.02)	na^f^	na^f^	-
Maternal BMI > 25 kg/m^2^	na^f^	-	24.1	1.01 (1.00, 1.02)	na^f^	na^f^	-
Maternal BMI > 30 kg/m^2^	na^f^	-	13.5	1.04 (1.02, 1.06) **	na^f^	na^f^	-
Birth weight < 2500 grams	1.0	1.02 (0.98, 1.07)	1.0	1.02 (0.97, 1.08)	0.549	0.4	1.08 (0.91, 1.29)
Birth weight < 10 percentile	7.5	1.00 (0.99, 1.02)	7.4	1.01 (0.99, 1.04)	0.687	6.1	1.03 (0.99, 1.08)
Birth weight > 90 percentile	11.4	1.03 (1.01, 1.04) **	11.2	0.98 (0.97, 1.00)	0.671	8.8	0.96 (0.93, 1.00) *
Birth weight > 4500 grams	4.0	1.04 (1.02, 1.07) **	3.9	0.95 (0.92, 0.98) **	0.530	2.5	0.93 (0.87, 0.99) *

^a^Variation between 16319–17848; ^b^Variation between 13785–15240; ^c^Variation between 4138–4214; ^d^ Age 21–37 years, normal weight (BMI 18.5-24.99), non-smoker, and without diabetes, preeclampsia, assisted reproduction, moderate/severe chronic disease, psychiatric disease, substance abuse, thromboembolic episode or known elevated risk, prior hypertension , induction of birth or caesarean section and difference between expected dates of delivery based on assessments from ultrasound and last menstrual period < 14 days; ^e^Simple linear regression analysis on year; ^f^Not applicable; ^g^Body mass index; ^h^Odds ratio from logistic regression analysis on year; ^i^Gestational age; ^j^Data on a period with increasing mean birth weight and proportion of large infants (1989–2000) are compared with those of a period with declining mean birth weight and proportions of large infants (2001–2010); * p < 0.05; ** p < 0.005.

The MFPR estimated BW increase was 27 grams prior to 2001/02 and the reduction was 45 grams thereafter (1989–2010 model, without PP-BMI and GWG). This MFPR model explained 23.5 % of the variance in BW.

From 2001/02 to 2009/10 mean (SD) PP-BMI increased from 24.36 (4.44) to 24.85 (5.02) kg/m^2^ (p<0.001), and mean (SD) GWG decreased from 14.79 (5.85) to 13.86 (5.79) kg (p<0.001). Adding these predictors and maternal height as exposure variables did not alter the declining trend in mean BW and rate of macrosomia. MFPR derived adjusted effect of YOB on BW was linear and negative, with an *estimated* (partial prediction) BW reduction of 51 grams (2001–2010 model, with PP-BMI and GWG). The adjusted odds ratio for macrosomia was 0.93 (95% CI: 0.90 to 0.96). The homogeneous subset did not differ in any essential ways from that of the whole cohort born during 2001–2010 (Table 1). The 2001–2010 MFPR models explained 35.2 % of the variance in BW for all and 32.8 % for the homogeneous subset.

Adjusted effects of significant exposure variables on BW are presented in Table 2, and the net effect (estimated from time trends) of significant predictors on change in BW from 2001/02 to 2009/10 are given in Table 3. Of the 50 grams *observed* reduction in mean BW from 2001/02 to 2009/10 the combined estimated net effect of the known individual level predictors explained 6 grams, leaving 44 grams unexplained.

**Table 2:** **Adjusted**^**a **^**effect of various exposure variables on birth weight in the regional (Oppland county) cohort**

	**Births 1989-2010**^**b**^**(n = 31070)**	**Births 2001-2010**^**c**^**(n = 13548)**	**Homogeneous subset**^**i**^**2001-2010**^**c**^**(n = 4136)**
**Predictors**^**d**^	**B**^**e**^**, grams (95% CI)**	**B**^**e**^**, grams (95% CI)**	**B**^**e**^**, grams (95% CI)**
Year of delivery (per year)	−1 ^f^ (−2, 0)	−7 (−4, -9)	−5 (−9, -1)
Mother’s age (per year)	−1 (−2, 0)	−1 (−3, 0)	−1 (−4, 2)
Mother’s height (per 1 cm)	na^g^	13 (12, 14)	13 (11, 15)
Pre-pregnancy BMI^h^ (per kg/m^3^)	na^g^	22 (21, 24)	26 (19, 32)
Gestational weight gain (per kg)	na^g^	18 (17, 19)	19 (16, 21)
Gestational age (per day)	25 (24, 25)	22 (21, 23)	22 (20, 23)
Male sex	104 (96, 115)	99 (86, 112)	78 (56, 100)
Multiparity	167 (156, 178)	181 (166, 195)	179 (155, 204)
Smoking	−171 (−183, -159)	−180 (−199, -160)	na^j^
Preeclampsia	−31 (−56, -6)	−81 (−113, -49)	na^j^
Diabetes	371 (326, 417)	298 (246, 350)	na^j^
Thromboembolism	−60 (−119, -1)	−97 (−155, -39)	na^j^
Prior premature birth	−96 (−141, -51)	−79 (−131, -27)	−81 (−169, 7)
Prior caesarean section	32 (10, 55)	39 (10, 67)	−94 (−171, -17)
Anaemia	82 (55, 110)	106 (77, 135)	97 (41, 152)
Oligohydramnion	−265 (−292, -238)	−257 (−284, -229)	−245 (−306, -184)
Polyhydramnion	299 (251, 347)	255 (205, 304)	282 (177, 386)

^a^Multiple linear regression analysis; ^b^Maternal prepregnant weight and gestational weight gain *not* included; ^c^Maternal prepregnant weight and gestational weight gain included; ^d^initially tested but non-significant predictors excluded from the final model: teenage pregnancy, marital status, occupational status, assisted reproductive technology, chronic disease, psychiatric disease, substance abuse, isolated hypertension in pregnancy, allergies, asthma, cervical conisation, haemoglobin > 13.5 gram/dl, bleeding in pregnancy, hyperemesis, infections in pregnancy, and a composite of complications related to placenta, foetal membranes, foetal distress, rhesus-immunisation; ^e^Change in birth weight (grams) per one unit change in predictor; ^f^Effect estimates vary from positive during 1989–2000 to negative during 2001–2010; ^g^Data not available before 2001; ^h^Body mass index; ^i^Age 21–37 years, normal weight (BMI 18.5-24.99), non-smoker, and without diabetes, preeclampsia, assisted reproduction, moderate/severe chronic disease, psychiatric disease, substance abuse, thromboembolic episode or known elevated risk, prior hypertension , induction of birth or caesarean section and difference between expected dates of delivery based on assessments from ultrasound and last menstrual period < 14 days; ^j^Not applicable.

**Table 3:** **Levels**^**a **^**of important predictors, and estimated effects of changes over time on birth weight (the regional cohort from 2001/2002 to 2009/2010, Oppland county)**

	**2001/2002**	**2009/2010**	**Effect on birth weight (grams)**^**b**^
	**mean (95% CI)**	**mean (95% CI)**	
PP-BMI^c^ , *kg/m*^*2*^	24.36 (24.19, 24.52)	24.85 (24.67, 25.03)	+11
GWG^d^, *kg* (mean, 95% CI)	14.79 (14.57, 15.01)	13.86 (13.65, 14.06)	−16
Gestational age, *days* (mean)	279.9 (279.6, 280.2)	279.2 (279.0, 279.5)	−15
	**prevalence %**	**prevalence %**	
Proportion smoking (%)	19	10	+16
Proportion diabetes^e^ (%)	1.0	2.6	+4
Proportion multiparity (%)	61.3	57.8	−6
**Total effect of above exposures**			**−6**

^a^For continuous variables: effect sizes from multiple regression analyses (Table 2) were multiplied by actual changes in mean values. For dichotomous variables: difference in number of exposed (calculated on basis of the mean number in 2009/2010, i.e. 1650) multiplied by the effect size of the exposure variable (Table 2), and divided by the mean number of births during 2009/2010; ^b^Estimated effect on difference in birth weight from 2001/2002 to 2009/2010; ^c^Pre-pregnancy body mass index; ^d^Gestational weight gain; ^e^Gestational diabetes included.

### National data on nutrition and physical activity

The reported national mean per capita daily caloric intake did not change during the study period; it was 2370 kcal in 1989–91, 2230 kcal in 1999–01 and 2250 kcal in 2007–09. Household consumption of main nutrients showed minimal variation. A small reduction in energy from carbohydrates (from 51% to 49%) was compensated by small increases in energy from protein (13% to 15%) and fat (35% to 36%). The annual per capita gross consumption (based on imports) of added sugars, included that of SSB, increased 5 kg to 45 kg during the 1990s and subsequently decreased 13 kg after year 2000, and the reported per capita daily energy contribution from sugar increased from 14 % in 1989–91 to 15 % in 1999–01, and decreased to 13 % in 2007–09.

Consumption of SSB showed a curve similar to that of the unadjusted and adjusted BW, with an increase and subsequent decrease of approximately 30 liters (3 kg sugar) per person per year (Figure 2). Consumption of other important individual or groups of food items either changed minimally (rice, eggs, cream, butter), or showed continuous increases (cereals, meat, cheese, vegetables, fruit juices, chocolate) or decreases (milk, margarine, potatoes) through the whole 22-year study period. None showed anything like the convex trend of sugar and SSB.

There was a steady increase in the proportion of 20–40 year old women who did leisure time physical activity more often than weekly, rising from 31.6 % in 1989 to 48 % in 1999 and 57.3 % in 2009. In the Norwegian population as a whole there was an increase in sedentary working conditions until last registration in 2002.

## Discussion

In this regional Norwegian cohort of infants born at term a temporary increase in mean BW, PI and proportion with macrosomia was not explained by changes in obstetric practice, maternal BMI, GWG, smoking habits, or morbidity during pregnancy. Nor could it be explained by changes in nutritional intakes or energy requirements as estimated from national consumer data, except possibly by a parallel change in consumption of sugar, in large part as SSB.

The strengths of this study were the population based design, almost complete participation, and consistent methods of obtaining data in the regional cohort. The weaknesses were the lack of data on maternal PPW and GWG prior to 2001, and lack of data on nutrition and physical activity during pregnancy. However, we suggest that estimates of changes in nutritional habits and physical activity from national surveys may be valid since the BW and macrosomia curves of this cohort were identical to those of all births in Norway [4,5] indicating that the studied cohort was representative of the Norwegian pregnancy population, and assuming that nutritional preferences of pregnant women did not differ significantly from those of the general population. The assumption that pregnant women consumed SSB similar to the national average is further supported by studies on Norwegian pregnant women during the studied period [12,13]. Furthermore, data on national consumption of food items and physical activity were consistently registered during the period [8] ([Norwegian Monitor 1989–2009, Synovate], Gunnar Breivik, personal communication, 2011).

Previous findings that increasing PP-BMI and GWG are associated with increasing BW and proportion of large newborns [17,18] were confirmed in the present study, but within the observed range their effects on BW were limited in the present study. Norwegian cohort studies have shown that women of reproductive age increased their BMI and obesity rate from the late 1980s till 2008 [19-21], and the mean PP-BMI of our cohort also increased from 2001 to 2010. It is therefore likely that PP-BMI increased continuously during 1989–2010 and therefore cannot explain the convex BW curve, although an estimated increase in BMI of 1.5-2.5 kg/m^2^ during the 1990’s, which has been the reported range of increase among women of childbearing age [19,20], may account for part of the unexplained increase in BW seen prior to year 2000. We have no reliable Norwegian data on GWG during 1989–2000. However, based on the small net modifying effect of this variable on the BW development during 2001–2010, it is unlikely that changes in GWG had a major influence.

The constant national consumption of energy and nationally stable composition of major nutrients during the study period suggest that the BW changes were not caused by changes in overall nutrition other than intakes of sugar, particularly from beverages. It is also unlikely that changes in physical activity, as seen nationally in women of fertile age, had a modifying effect on nutritional balance in a way fitting the BW curve. We therefore suggest that the development of mean BW and proportion with macrosomia was largely due to changes in consumption of rapidly absorbable sugar, and that the effect of sugar was more related to sugar consumption *per se* than through changes in maternal PPW and GWG. The effect of 44 grams unexplained change in mean BW in our regional cohort may seem small, but considering a cohort effect and the large difference in proportion with macrosomia, which may possibly reflect an uneven distribution of sugar consumers among pregnant women or different sensitivity on part of the fetus to the effect of sugar, the potential significance for later OWOB may be substantial.

Increasing BW and proportion of large newborns until around 2000 [17,22-27] and subsequent leveling-off or decrease [24,25,28-31] have been reported from several countries, e.g. the US, UK, Australia, Germany and Sweden. Increasing maternal PP-BMI, GWG, and rates of gestational diabetes, and lower smoking rates have been suggested as causes for increase [17,26,32], but the recent leveling-off or decreases in BW parameters have occurred despite reports of unchanged trends in these exposures [25,28,30]. Suggested causes of lack of further increases in BW parameters have been that large fetuses tend to result in earlier spontaneous delivery [33,34], and that changes in obstetric practices have affected BW trends, e.g. through reduced GA due to frequent induction of labor and elective caesarean section in general [23,25,28,29], in obese women [35], when large babies are expected [36], or to avoid postmaturity [28,37]. In the present study the effect of decreasing GA on BW was adjusted for without altering the overall trend in BW.

The notion that consumption of rapidly absorbed sugar may increase BW may be supported by similarities of BW patterns and reported SSB consumption patterns in other countries, such as the US, UK, Australia and Sweden [38-41], and by reports that sugar from SSB has contributed significantly to total energy intake in women of reproductive age [13,42,43]. A small study has suggested that diets during pregnancy with a high glycemic index may result in increased BW [44] although no conclusion on this issue could be drawn in a recent systematic review [45]. Whether intake of rapidly absorbed sugar may have a specific effect on BW, either through changes in maternal weight or directly on the fetus, has to our knowledge not been addressed, although such an interpretation may be surmised from a recent study [46].

Studies in non-diabetics have shown a positive relationship between fasting and non-fasting blood glucose levels and frequencies of blood glucose peaks during pregnancy and BW, macrosomia and neonatal adiposity [47-50]. SSB contain sucrose or high-fructose corn-syrup, and may cause rapid and marked increases in blood glucose [51,52]. High or frequent intakes may particularly cause hyperglycemia in subjects with reduced insulin sensitivity, such as during pregnancy [53]. Increased blood glucose in the mother, and therefore also the fetus, causes increased fetal insulin response [54] and increased fetal growth since insulin is an important fetal growth hormone [55]. Additionally, postprandial glucose peaks in the mother may possibly result in glucose peaks higher than the renal threshold in the fetus leading to increased glucose in amniotic fluid and enhanced insulin production from prolonged intestinal absorption of ingested amniotic fluid [56]. The fructose content in SSB may also contribute to increased BW by causing increased levels of plasma triglycerides (TG) [52] and elevated plasma glucose because of reduced hepatic insulin sensitivity [57]. TGs are transferred to the fetus, and maternal levels correlate with BW [58].

It is intriguing that BW in our region and in Norway started to increase at the time when large recappable soft drink bottles, which may encourage frequent and large intakes, became available in Norway [59], that mean BW and proportion of large babies increased and decreased in parallel to the national consumption of sugar and SSB in a way that could not be reliably explained by individual level effects on maternal weight in our regional cohort, and that the average increase in national sugar consumption during the period of increasing BW largely could be explained by the increasing use of SSB. The reduction in sugar consumption based on gross national sugar imports was larger after 2000 (13 kg) than the increase during the 1990s (5 kg). These changes were estimates from data obtained 5–10 years apart and contain a number of acknowledged uncertainties, e.g. in relation to size of a national emergency storage [8,60]. These estimates are, therefore, less reliable with respect to yearly consumption than estimates based on annual surveys of a representative proportion of the Norwegian population by Statistics Norway which did not show dramatic changes in sugar intakes, and the annual sales of SSB which were parallel to the BW curve.

## Conclusion

In our regional cohort the known predictors of BW could not explain the observed temporal changes in BW and macrosomia, and on basis of the observed national time trends in nutrition and physical activity we therefore suggest that increased fetal growth may at least partly be explained by a direct effect of rapidly absorbable sugar, largely from high and frequent intakes of SSB. If true, the suggested effect of sugar on fetal and possibly later health may be of great concern, and this issue needs to be explored more specifically, e.g. through large cohort studies where detailed data on nutrition and physical activity during pregnancy are included. Diets rich in fiber and low in added sugar may contribute to glycemic control [61]. Whether such or other diets pertaining to avoid excessive blood glucose levels may be of benefit for fetal growth ought to be explored.

## Abbreviations

BW: birth weight; PP-BMI: pre-pregnancy body mass index; GWG: gestational weight gain; SD: standard deviation; SSB: sugar-sweetened beverages; OWOB: overweight and obesity; MBRN: Medical Birth Registry of Norway; EDD: estimated date of delivery; PPW: pre-pregnancy weight; PI: ponderal index; CI: confidence intervals; OR: odds ratio; MFPR: multiple fractional polynomial regression; YOB: year of birth; TG: triglycerides.

## 

The authors have not published or submitted any related papers from this study.

## Competing interests

The author(s) declare that they have no competing interests. All authors have completed and submitted the ICMJE Form for Disclosure of Potential Conflicts of Interest, and none were reported.

## Additional contributions

We thank Gunnar Breivik, Professor, Department of Cultural and Social Studies, Norwegian School of Sport Sciences, for providing data on recreational physical activity in women of reproductive health (personal communication).

## Authors’ contributions

JHG participated in drafting the study concept and design, performed statistical analyses and interpretation of data, and drafted and critically revised the manuscript. TM participated in drafting the study concept and design, analyses and interpretation of data, and drafting and revising the manuscript. JN was responsible for acquisition of data, participated in drafting the study concept and design, analyses and interpretation of data, and revising of the manuscript. GEE participated in statistical analysis and interpretation of data, and revision of the manuscript. All authors read and approved the final manuscript.

## Funding

This study was supported by an unrestricted grant from the South-Eastern Norway Regional Health Authority. The funding source did not play any role in the design and conduct of the study; in the collection, management, analysis, or interpretation of the data; or in the preparation, review, or approval of the manuscript.

## Pre-publication history

The pre-publication history for this paper can be accessed here:

http://www.biomedcentral.com/1471-2458/12/901/prepub
